# Comparative Proteomics Analysis of Human Macrophages Infected with Virulent *Mycobacterium bovis*

**DOI:** 10.3389/fcimb.2017.00065

**Published:** 2017-03-09

**Authors:** Pei Li, Rui Wang, Wenqi Dong, Linlin Hu, Bingbing Zong, Yanyan Zhang, Xiangru Wang, Aizhen Guo, Anding Zhang, Yaozu Xiang, Huanchun Chen, Chen Tan

**Affiliations:** ^1^State Key Laboratory of Agricultural Microbiology, College of Veterinary Medicine, Huazhong Agricultural UniversityWuhan, China; ^2^Key Laboratory of Development of Veterinary Diagnostic Products, Ministry of Agriculture, Huazhong Agricultural UniversityWuhan, China; ^3^Advanced Institute of Translational Medicine, School of Life Sciences and Technology, Tongji UniversityShanghai, China

**Keywords:** *M*. *tb*, *M. bovis*, THP-1 cell, iTRAQ, proteomics, pathway analysis

## Abstract

*Mycobacterium bovis* (*M. bovis*), the most common pathogens of tuberculosis (TB), is virulent to human and cattle, and transmission between cattle and humans warrants reconsideration concerning food safety and public health. Recently, efforts have begun to analyze cellular proteomic responses induced by *Mycobacterium tuberculosis* (*M. tb*). However, the underlying mechanisms by which virulent *M. bovis* affects human hosts are not fully understood. For the present study, we utilized a global and comparative labeling strategy of isobaric tag for relative and absolute quantitation (iTRAQ) to assess proteomic changes in the human monocyte cell line (THP-1) using a vaccine strain and two virulent strains *H37Rv* and *M. bovis*. We measured 2,032 proteins, of which 61 were significantly differentially regulated. Ingenuity Pathway Analysis was employed to investigate the canonical pathways and functional networks involved in the infection. Several pathways, most notably the phagosome maturation pathway and TNF signaling pathway, were differentially affected by virulent strain treatment, including the key proteins CCL20 and ICAM1. Our qRT-PCR results were in accordance with those obtained from iTRAQ. The key enzyme MTHFD2, which is mainly involved in metabolism pathways, as well as LAMTOR2 might be effective upon *M. bovis* infection. String analysis also suggested that the vacuolar protein VPS26A interacted with TBC1D9B uniquely induced by *M. bovis*. In this study, we have first demonstrated the application of iTRAQ to compare human protein alterations induced by virulent *M. bovis* infections, thus providing a conceptual understanding of mycobacteria pathogenesis within the host as well as insight into preventing and controlling TB in human and animal hosts' transmission.

## Introduction

Tuberculosis (TB) is an infectious disease that greatly impacts human and animal health worldwide. TB is caused by members of the *Mycobacterium tuberculosis (M. tb)* complex (MTBC), of which *M. tb* is a primary causative agent in humans. A recent report on global tuberculosis from the World Health Organization (WHO) indicated that 10.4 million people became ill and that 1.8 million died from TB in 2015 (World Health Organization, 2016). In a study in which cattle were artificially infected with two *M. tb* strains, it was confirmed that *M. tb* is virulent to cattle (Chen et al., 2013). Tuberculosis in animals is primarily observed in cattle and other bovids, for which the disease is generally referred to as bovine tuberculosis (bTB) and is mainly caused by *Mycobacterium bovis* (*M. bovis*). Animal tuberculosis bears a zoonotic potential and is therefore a public health concern (Cosivi et al., 1998; Renwick et al., 2007; Michel et al., 2010; Koul et al., 2011). Thus, *M. tb and M. bovis* are the most common pathogens within the MTBC (Ernst et al., 2007). Although *M. bovis* is the main causative agent of bTB, it has the broadest host range among all MTBC members (Meikle et al., 2011). Globally, most cases of zoonotic TB caused by *M. bovis* have been anecdotally reported, including TB in humans that is usually associated with extrapulmonary TB (Ayele et al., 2004; Müller et al., 2013; Gallivan et al., 2015; Jiang et al., 2015; El-Sayed et al., 2016). *M. bovis* resulted from genomic evolutionary reduction of *M. tb*, although the two species share 99.95% genomic identity (Garnier et al., 2003; Bigi et al., 2016). Since 1921, *M. bovis* bacillus Calmette-Guérin (BCG) has been recognized as an important and available vaccine to prevent tuberculosis worldwide (Mahairas et al., 1996). It is an attenuated strain of *M. bovis* developed by Calmette and Guérin, which lost its virulence after 230 passages over a period of 13 years (Hsu et al., 2003; Seki et al., 2009). As *M. bovis* causes zoonotic diseases (Dye and Williams, 2010), it is necessary to have a conceptual and clear understanding of mycobacteria transmission dynamics and pathogenesis within populations and between hosts, thus providing insight into better improvement of TB control through novel and collaborative research and public health efforts.

Macrophages (MΦs) are the primary effector cells responsible for killing *M. tb* through various mechanisms, including the induction of toxic anti-microbial effectors, stimulation of microbe intoxication mechanisms, apoptosis, lipid mediators, microRNAs, and cytokines (van crevel et al., 2002; Rajaram et al., 2014; Weiss and Schaible, 2015). However, MTBC can evade host immunity to create a favorable environment for intracellular replication via their lipids, inhibition of phagosome-lysosome fusion and phagosome acidification, hijacking host cell signaling, downregulation of host gene expression, and formation of granuloma (Cosma et al., 2003; Rohde et al., 2007; Davis and Ramakrishnan, 2009; Meena, 2010; Arbues et al., 2015; Chandran et al., 2015; Colonne et al., 2016). Therefore, quantitative and comparative proteomic analyses have been used to provide complementary information about host responses to MTBC infection, particularly *M. tb* (Giri et al., 2010; Schmidt and Völker, 2011; He et al., 2012; Jamwal et al., 2013; Kunnath-Velayudhan and Porcelli, 2013; Petriz and Franco, 2014). Researchers have identified eight intraphagosomal expressed proteins of the BCG strain during infection with macrophages (Singhal et al., 2016). Such proteomic data have provided enhanced characterization of MTBC and host-derived targets to better improvement of TB control. While the vast majority of proteomic research about virulent *M. bovis*-host interaction focused on biomarkers during infection of cattle, MTBC natural reservoir hosts, such as the Eurasian wild boar (*Sus scrofa*), and clinical specimens have also been assessed (Naranjo et al., 2007; Garton et al., 2008; Seth et al., 2009; Blanco et al., 2014; Lamont et al., 2014; Young et al., 2014; Esterhuyse et al., 2015; López et al., 2016).

*M. bovis* is responsible for great economic losses and cattle-to-human transmission and represents a severe threat to public health (Cosivi et al., 1995; Fogel, 2015). Since the pathogenesis of bTB is not as well understood as human tuberculosis, we employed proteomic analyses by examining activated THP-1 cells infected with the vaccine strain BCG and two virulent MTBC strains, including *M. tb*-*H37Rv* and *M. bovis*, by isobaric tag for relative and absolute quantitation (iTRAQ). We identified and measured 2,032 proteins with two or more peptides at >99% confidence and found that 61 were significantly regulated. There also were major differences in the proteins and pathways induced by the virulent MTBC strains when compared to the vaccine strain BCG. *H37Rv* and *M. bovis* both induced significant up-regulation of several proteins in the TNF signaling pathway. Immunological disease, as well as inflammatory response and disease, were more prominent upon infection of cells with virulent *H37Rv* and *M. bovis* than the BCG strain. This work serves as an enhancement of the understanding that *M. bovis* affects proteins obtained from human host cells. These results may provide useful insights in understanding MTBC pathogenesis, particularly *M. bovis*, revealing novel biomarkers that may be critical to TB cattle-to-human transmission and diagnosis. Such foundational knowledge we have obtained from this study may drive more efficacious treatment and hopefully eventual eradication of TB.

## Materials and methods

### Bacterial strains and culture conditions

The *M. tb* reference strain *H37Rv* (ATCC 27294), BCG Tokyo strain (ATCC 35737), and *M. bovis* (ATCC 19210) were kindly provided by Dr. Chuanyou Li, Beijing Tuberculosis & Thoracic Tumor Research Institute (Beijing, China). All strains were cultured to mid-log phase in Middlebrook 7H9 medium (Becton Dickinson and Company, Franklin Lakes, NJ, USA) supplemented with 10% oleic acid, albumin, dextrose, and catalase medium (OADC) (Becton Dickinson and Company) and 0.05% Tween 80 (Amresco Inc., Solon, OH, USA) with agitation. Cultures were maintained in a biosafety level 3 facility at Huazhong Agricultural University (Wuhan, China) and stored at −80°C. Bacteria were harvested from the culture medium by centrifugation at 4,000 g for 10 min washed once in 1,640 medium, resuspended in 1,640 to an OD600 of 1, equivalent to 3 × 10^8^ bacteria/ml. Before measuring the absorbance at 600 nm, a syringe connected to a sterile needle was used to scatter the bacterial clumps.

For the colony counting assay, bacteria were serially diluted 10-fold with Middlebrook's 7H11 medium and 0.1 ml of each dilution was transferred to 7H11 plates. Bacteria were grown for about 4 weeks until the colonies were suitable for counting by eye. Bacterial concentration was recorded as c.f.u. ml^−1^. CFUs were calculated from the actual colony counts obtained to guarantee the same initial infection dose. Detailed protocols and culture conditions are described elsewhere (Kumar et al., 2010; Wassermann et al., 2015).

### Cell culture and infection

The human monocyte cell line THP-1 was cultured in RPMI 1,640 supplemented with 2 mM L-glutamine and 10% FBS (Hyclone, GE Healthcare Life Science, Grand Island, USA). THP-1 cells were activated using 100 ng/mL phorbol myristate acetate (PMA) and incubated at 37°C in a 5% CO_2_ atmosphere for 48 h. To reduce the effect of PMA from THP-1 cells, the medium was replaced, and incubation was continued for another 8 h. They were then infected with *H37Rv, M. bovis*, or BCG at an MOI of 10 (10 bacteria to one cell) (Hmama et al., 1998; Chen et al., 2013; Zhang et al., 2013; Wassermann et al., 2015). After 24 h, cells were collected, washed three times with ice-cold phosphate-buffered saline (PBS) and processed for iTRAQ analysis or total RNA extraction. Three independent experiments were performed.

### Protein preparation, protein digestion, and iTRAQ labeling

The MTBC-infected and mock-infected cells in 10 cm cell culture flasks were collected using cell scrapers and suspended in 400 μL lysis buffer containing 8 M urea, 30 mM HEPES, 1 mM PMSF, 2 mM EDTA, and 10 mM DTT. Further lysate debris was moved by centrifugation at 20,000 g for 30 min at 4°C after ultrasonication treatment for 5 min (pulse on: 2 s and pulse off: 3 s; power: 180 W). The supernatants were reduced with 10 mM DTT at 56°C for 1 h and alkylated with 55 mM IAM in the darkroom for 1 h. The protein mixtures were precipitated by adding 4 × volume of chilled acetone at −20°C overnight. After centrifugation at 20,000 g for 30 min at 4°C, the pellet was dissolved in 0.5 M TEAB (Applied Biosystems, Milan, Italy) with 0.1% SDS via an ultrasonication treatment for 5 min. After centrifuging at 20,000 g for 30 min at 4°C, an aliquot of the supernatant was taken to determine protein concentration via the Bradford assay (Sangon Biotech, Shanghai, China).

For each sample, total protein (100 μg) was digested with 3.3 μl of trypsin (1 μg/μl) (Promega, Madison, WI, USA) at 37°C for 24 h. After trypsin digestion, peptides were dried via vacuum centrifugation. Peptides were reconstituted in 0.5 M TEAB and processed according to the manufacturer's instructions (Applied Biosystems). Briefly, one unit of iTRAQ reagent (AB Sciex, Foster City, CA, USA) was thawed and reconstituted in 24 μL isopropanol. Samples were labeled with the iTRAQ tags as follow: BCG-infected samples (tag 115), *H37Rv*-infected samples (tag 116), *M. bovis*-infected samples (tag 117), and mock-infected samples (tag 118). The peptides were then incubated at room temperature for 2 h. The labeled samples were then mixed and dried with a rotary vacuum concentrator.

### LC-MS/MS analysis

Strong cation exchange (SCX) chromatography (Phenomenex, USA) was performed with an LC-20AB HPLC pump system (Shimadzu, Kyoto, Japan) to separate samples. The iTRAQ-labeled peptide mixtures were reconstituted and dissolved in buffer A (10 mM KH_2_PO_4_ in 25% ACN, pH 3.0) and loaded onto a 4.6 × 250 mm Ultremex SCX column containing 5 μm particles (Phenomenex). The peptides were eluted at a flow rate of 1 mL/min with a gradient of buffer A for 30 min, 5–50% buffer B (10 mM KH_2_PO_4_ and 2 M KCl in 25% ACN, pH 3.0) for 27 min, and 60–100% buffer B for 15 min. The system was then maintained at 100% buffer B for 1 min before equilibrating with buffer A for 10 min prior to the next injection. The eluted peptides were desalted with a Strata X C18 column (100 × 75 mm; 5 μm particles; 300 Å aperture; Phenomenex, USA) and vacuum dried.

The fractions above were dissolved in aqueous solution containing 0.1% FA and 2% ACN and then centrifuged at 12,000 g for 10 min at 4°C. Five micrograms supernatant was loaded on an LC-20AD nano HPLC (Shimadzu, Kyoto, Japan) by the auto sampler onto a 2 cm C18 trap column (inner diameter 200 μm, Waters), and the peptides were eluted onto a resolving 10 cm analytical C18 column (inner diameter 75 μm, Waters). The mobile phases used were composed of solvent A (0.1% FA and 5% ACN) and solvent B (0.1% FA and 95% ACN). The gradient was run at 400 nL/min for 48 min at 5–80% solvent B, followed by running a linear gradient to 80% for 7 min, maintained at 80% B for 3 min, and finally returned to 5% in 7 min. The peptides were subjected to nano electrospray ionization followed by tandem mass spectrometry (MS/MS) in a Q EXACTIVE (Thermo Fisher Scientific, San Jose, CA, USA) coupled to the HPLC. Intact peptides were detected in the Orbitrap at a resolution of 70,000 and a mass range of 350−2,000 m/z. Peptides were selected for MS/MS using high-energy collision dissociation (HCD), and ion fragments were detected in the Orbitrap at a resolution of 17,500. The electrospray voltage applied was 1.8 kV. MS/MS analysis was required for the 15 most abundant precursor ions, which were above a threshold ion count of 20,000 in the MS survey scan, including a following dynamic exclusion duration of 15 s.

### Bioinformatics and data analysis

Raw data files acquired from the mass spectrometers were converted into MGF files using 5,600 MS converter, and the MGF files were searched. Protein identification was performed using the Mascot search engine (Matrix Science, London, UK; version 2.3.0) against the Uniprot _2015_human database (containing 20,194 sequences). For protein identification, a mass tolerance of 15 ppm was permitted for intact peptide masses and 0.05 Da for fragmented ions, with allowance for one missed cleavage in the trypsin digests. Gln → Pyro-Glu (N-term Q), oxidation (M), and deamidated (NQ) were potential variable modifications and carbamidomethyl (C), iTRAQ 8plex (K), iTRAQ 8plex (Y), iTRAQ 8plex (N-term) were fixed modifications. Specifically, an automatic decoy database search was performed in Mascot by choosing the decoy checkbox in which a random sequence of the database is generated and tested for raw spectra as well as the real database. To reduce the probability of false peptide identification, only peptides with significance scores (≥20) at the 99% confidence interval as determined by a Mascot probability analysis were included. In addition, each confident protein identification involved at least one unique peptide. The quantitative protein ratios were weighted and normalized by the median ratio in Mascot. Statistical significance analyses were evaluated using two-way ANOVA. For comparison between samples, a protein with a ratio of mean fold change >1.2 (or <0.83) with an Exp pr > 0.05 and a Group pr < 0.05 was regarded as differentially expressed (Exp pr, three-experiment *p*-value; Group pr, group *p*-value; Fold Change = Experiment + Group + Error).

Gene Ontology (GO) analysis was conducted using the Blast2GO program against the non-redundant protein database, and function characterization, biological network, pathway analysis were all performed using the Ingenuity Pathway Analysis software (IPA, http://www.ingenuity.com). The probable interacting partners were predicted using the STRING-10 server (http://string.embl.de/). STRING is a database of both known and predicted protein-protein interactions. These include direct (physical) and indirect (functional) associations, which are derived from four separate sources: genomic context, high-throughput experiments, co-expression, and prior knowledge. A network was made at a medium confidence level (0.400), allowing all active prediction methods (Sharma et al., 2016).

### RNA extraction and real-time PCR analysis

Total cellular RNA was extracted from MTBC-infected and mock-infected THP-1 cells using the TRIzol reagent (Invitrogen) according to the manufacturer's protocol. After the RNA was reverse-transcribed to first-strand cDNA using oligo (dT) as the primer (Invitrogen), quantitative real-time PCR was performed using an Applied Biosystems ViiA 7 real-time PCR system. Reactions were each 10 μl containing 50 ng of cDNA, 2 × SYBR Green Master Mix Reagent (Applied Biosystems), and 100 nM of gene-specific primers. The thermocycling conditions were 95°C for 5 min, and 40 cycles at 95°C for 30 s, 60°C for 30 s, and 72°C for 30 s. Amplification specificity was assessed using melting curve analyses. Transcript abundances were normalized to GAPDH, which served as the internal control. The primers used are listed in Table S1. The degree of expression change was calculated using the 2^−ΔΔCt^ method. Each cDNA sample was amplified in triplicate. The data analysis was performed also using the Applied Biosystems ViiA 7 real-time PCR system software (Applied Biosystems).

### Statistical analysis

All statistical analyses were performed using the GraphPad Prism software (version 6.02). Values are showed as mean ± standard deviation (SD), *p* < 0.05 was considered statistically significant.

## Results

**Figure 1:**
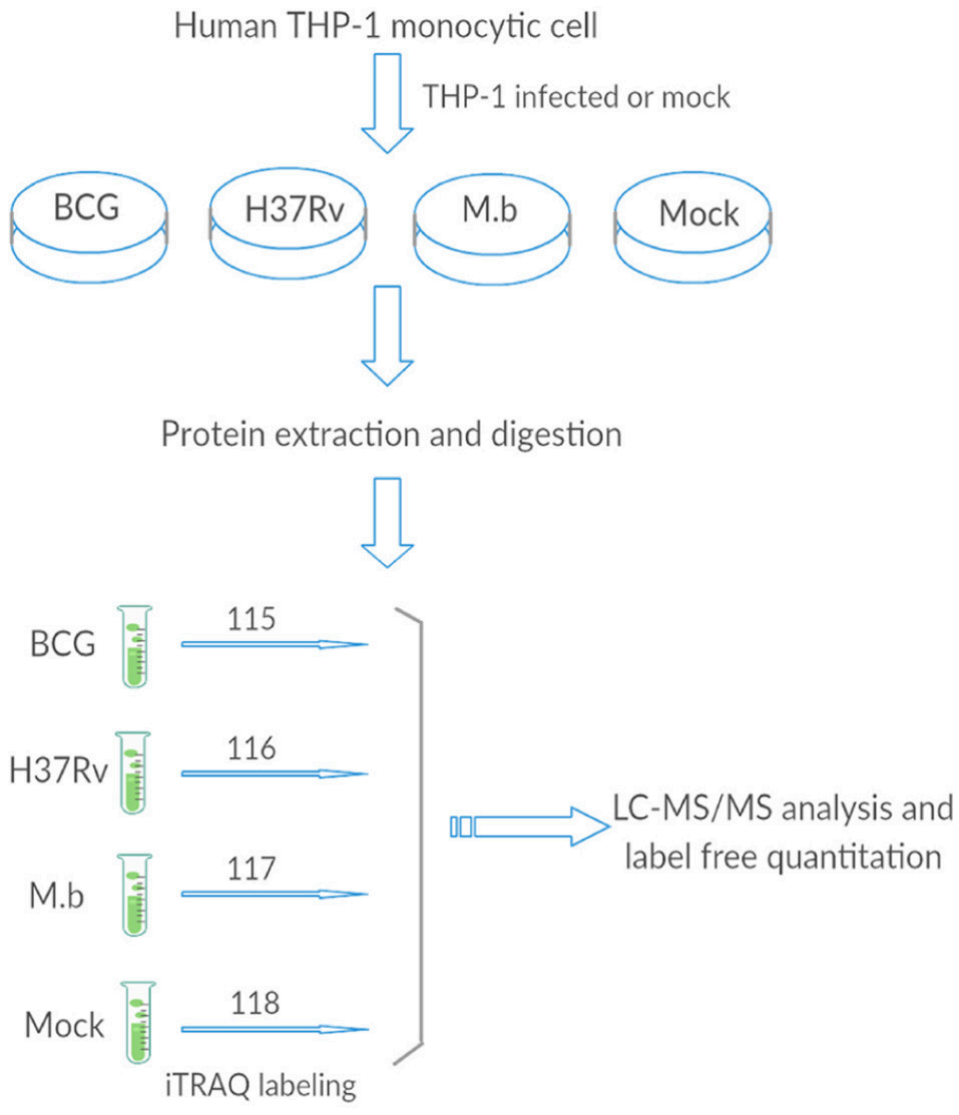
**The general work flow**.

We employed label-free quantitative proteomics to analyze whole cell lysates harvested from THP-1 infected with either *M. bovis, M. tb*-*H37Rv*, or BCG strains, and biological triplicates were performed (Figure 1). We quantified 2,032 proteins in each group, resulting in a total of 15,139 unique peptides from these 12 samples (we mock-infected cells also), however, 283 unique peptides were not detected in this study (Table S2).

### *M. bovis, H37Rv*, and BCG induce different host protein regulation

**Figure 2:**
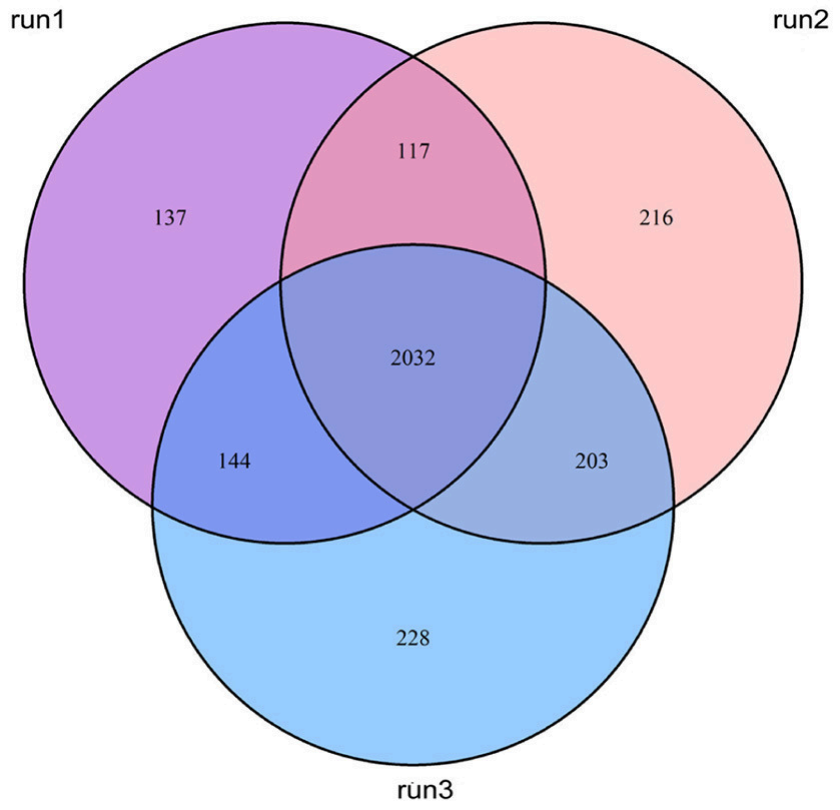
**Venn diagrams show the overlap of quantified proteins**.

**Figure 3:**
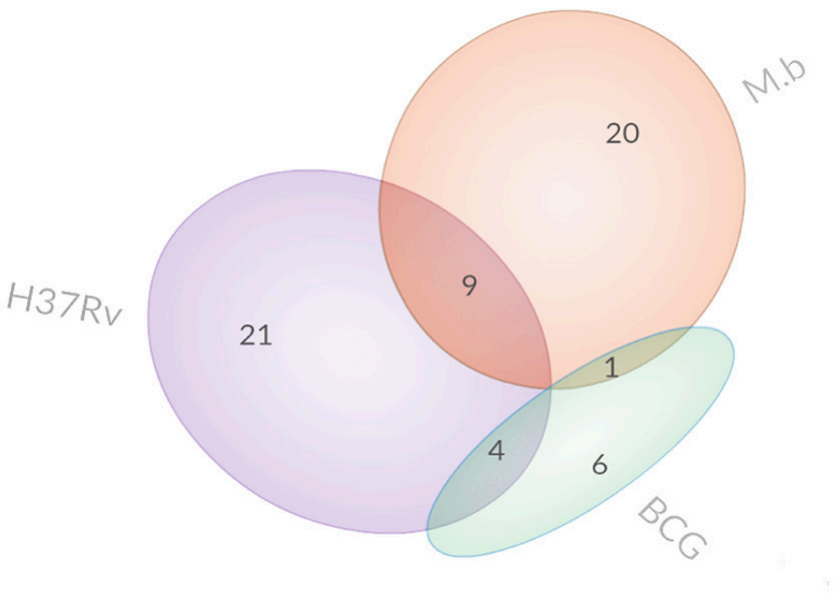
**Venn diagrams display the overlap of differentially expressed proteins**.

In this study, we employed the use of human monocyte THP-1 cells after first differentiating them with PMA. The iTRAQ data showed that a total of 2,481, 2,569, and 2,608 proteins were quantified for three replicates, which were expressed as run 1, run 2, and run 3, respectively (Figure 2). Among them, 2,032 proteins were common in all three biological replicates. Significant up/down regulations between samples were determined by a mean fold change >1.2 (or <0.83) with an Exp pr > 0.05 and a Group pr < 0.05 for peptide quantification. In total, 61 proteins were significantly up or down-regulated during MTBC infection (Figure 3).

Specifically, 26 proteins were up-regulated and 4 proteins were down-regulated during *M. bovis* infection. Meanwhile, 23 proteins were up-regulated and 11 proteins were down-regulated during *H37Rv* infection; 9 proteins were up-regulated and 2 proteins were down-regulated during BCG strain infection. In comparison with the uninfected cells, there was no common protein among those our MTBC-infected groups (Table 1).

**Table 1:** **Significantly regulated THP-1 cell proteins**.

**Accession**	**Gene symbol**	**Protein name**	**Mean of iTRAQ radio**			**Unique peptide**			**Mascot score**		
			**BCG**	***H37Rv***	***M. bovis***	**Run 1**	**Run 2**	**Run 3**	**Run 1**	**Run 2**	**Run 3**
P09914	IFIT1	Interferon-induced protein with tetratricopeptide repeats 1	–	1.638^***^	–	5	5	4	148.49	184.93	87.36
P04179	SOD2	Superoxide dismutase [Mn], mitochondrial	–	1.625^***^	1.247^***^	5	7	6	236.95	271.18	275.14
P35527	KRT9	Keratin 9	–	1.573^***^	1.629^***^	3	6	5	99.83	246.23	219.89
P78556	CCL20	C-C Motif Chemokine Ligand 20	–	1.522^*^	1.419^**^	1	1	2	28.16	42.07	51.06
O14879	IFIT3	Interferon Induced Protein With Tetratricopeptide Repeats 3	–	1.457^***^	–	3	4	5	198.26	117.87	186.89
P02656	APOC3	Apolipoprotein C3	–	1.393^***^	–	1	1	1	77.33	74.64	70.12
P27449	ATP6V0C	ATPase H+ Transporting V0 Subunit C	1.382^**^	–	–	1	1	2	23.41	22.74	41.17
P05362	ICAM1	Intercellular Adhesion Molecule 1	–	1.373^***^	1.238^***^	8	8	6	255.22	297.17	199.31
P14598	NCF1	Neutrophil Cytosolic Factor 1	–	1.353^***^	1.291^***^	4	4	4	128.34	100.99	147.69
P03905	MT-ND4	Mitochondrially Encoded NADH:Ubiquinone Oxidoreductase Core Subunit 4	1.249^*^	1.332^*^	–	2	2	2	55.52	61.29	73.14
P13805	TNNT1	Troponin T1, Slow Skeletal Type	–	–	1.323^***^	1	1	1	16.47	30.67	35.92
P05452	CLEC3B	C-Type Lectin Domain Family 3 Member B	–	1.321^***^	–	2	1	1	52.25	34.57	21.26
O75436	VPS26A	VPS26, Retromer Complex Component A	–	–	1.319^***^	3	3	3	97.51	71.29	96.09
P02649	APOE	Apolipoprotein E	–	1.307^***^	1.262^***^	2	1	2	40.52	24.21	40
Q13501	SQSTM1	Sequestosome 1	–	1.304^***^	–	1	2	2	47.62	77.16	87.02
P02794	FTH1	Ferritin Heavy Chain 1	1.292^***^	–	–	3	3	5	80.37	91.06	138.24
Q03405	PLAUR	Plasminogen Activator, Urokinase Receptor	–	1.286^***^	–	5	4	5	209.48	165.44	210.38
Q96I25	RBM17	RNA Binding Motif Protein 17	–	–	1.282^*^	1	2	1	48.45	79.29	28.82
Q6RW13	AGTRAP	Angiotensin II Receptor Associated Protein	–	–	1.273^***^	2	2	2	61.58	63.29	69.08
P84098	RPL19	Ribosomal Protein L19	1.301^***^	1.270^***^		5	4	4	76.23	81.06	85.81
Q9NY65	TUBA8	Tubulin Alpha 8	1.253^**^	–		1	1	1	681.78	688.55	712.14
P01344	IGF2	Insulin Like Growth Factor 2	–	1.256^***^		1	1	1	42.78	40.83	41.24
Q6BDI9	REP15	RAB15 Effector Protein	–	–	1.255^***^	1	1	1	61.5	61.61	61.81
Q13459	MYO9B	Myosin IXB	–	1.253^*^		2	6	5	62.97	153.08	115.88
Q9NX76	CMTM6	CKLF Like MARVEL Transmembrane Domain Containing 6	–	–	1.254^***^	1	1	1	38.65	47.94	42.54
P61619	SEC61A1	Sec61 Translocon Alpha 1 Subunit	1.243^**^	–	–	7	8	6	243.58	322.02	209.66
P30613	PKLR	Pyruvate Kinase, Liver And RBC	–	–	1.243^***^	1	1	1	130.59	125.43	115.26
Q9Y2Z4	YARS2	Tyrosyl-TRNA Synthetase 2	–	–	1.233^***^	2	3	3	116.14	132.18	113.12
Q96CT7	CCDC124	Coiled-Coil Domain Containing 124	–	–	1.232^**^	2	3	3	67.78	95.98	119.15
O14531	DPYSL4	Dihydropyrimidinase-related protein 4	–	–	1.223^*^	1	1	1	29.77	41.49	32.94
Q9Y5U9	IER3IP1	Immediate Early Response 3 Interacting Protein 1	–	1.227^*^	–	1	2	2	33.62	59.77	81.24
P09110	ACAA1	Acetyl-CoA Acyltransferase 1	–	–	1.222^***^	3	4	5	105.43	69.91	140.44
Q96ER9	CCDC51	Coiled-Coil Domain Containing 51	–	–	1.220^***^	1	1	2	22.68	32.53	60.1
P49207	RPL34	Ribosomal Protein L34	1.223^***^	–	–	4	3	2	93.66	73.5	77.46
P13995	MTHFD2	Methylenetetrahydrofolate Dehydrogenase (NADP+ Dependent) 2, Methenyltetrahydrofolate Cyclohydrolase	–	–	1.219^***^	2	2	3	60.96	61.87	104.25
P26373	RPL13	Ribosomal Protein L13	1.222^***^	1.216^***^	–	9	9	9	286.35	313.93	319.6
Q07065	CKAP4	Cytoskeleton Associated Protein 4	–	–	1.213^**^	1	1	4	23.45	31.9	97.7
P62304	SNRPE	Small Nuclear Ribonucleoprotein Polypeptide E	–	1.210^*^	–	3	2	2	97.86	61.16	73.07
Q9BUN8	DERL1	Derlin 1	–	1.207^**^	1.211^***^	2	3	3	43.42	74.08	74.96
Q567U6	CCDC93	Coiled-Coil Domain Containing 93	–	–	1.204^*^	1	3	3	27.14	81.94	79.91
Q92597	NDRG1	N-Myc Downstream Regulated 1	–	–	1.204^***^	3	3	4	104.71	124.65	160.12
O75396	SEC22B	SEC22 Homolog B, Vesicle Trafficking Protein	–	1.207^***^	–	7	8	7	365.95	375.06	340.9
P43003	SLC1A3	Solute Carrier Family 1 Member 3	–	1.205^*^	–	1	1	1	23.42	34.99	22.09
Q9Y2Q5	LAMTOR2	Late Endosomal/Lysosomal Adaptor, MAPK And MTOR Activator 2	1.224^*^	–	1.203^**^	1	2	2	58.18	77.29	65.55
Q66K14	TBC1D9B	TBC1 Domain Family Member 9B	–	–	1.202^*^	3	4	5	123.35	94.66	144.47
P18124	RPL7	Ribosomal Protein L7	–	1.202^***^	–	9	11	12	251.33	285.77	295.58
Q5VTR2	RNF20	Ring Finger Protein 20	–	–	1.201^***^	1	2	1	49.3	49.3	61.94
P09382	LGALS1	Galectin 1	–	0.830^***^	–	9	10	10	410.99	415.65	428.47
Q9Y2S6	TMA7	Translation Machinery Associated 7 Homolog	–	–	0.827^*^	1	1	1	28.34	26.84	31.78
Q9P1F3	ABRACL	ABRA C-Terminal Like	–	0.820^***^	–	3	1	4	72.78	44.92	123.6
Q96CX2	KCTD12	Potassium Channel Tetramerization Domain Containing 12	–	0.815^***^	–	2	3	1	63.74	105.36	25.95
P07858	CTSB	Cathepsin B	–	0.814^***^	–	7	7	8	290.13	291.36	308.38
P58546	MTPN	Myotrophin	–	–	0.812^***^	2	2	1	80.51	103.9	61.27
P60520	GABARAPL2	GABA Type A Receptor Associated Protein Like 2	–	0.808^***^	–	1	1	1	47.83	34.39	26.14
Q01469	FABP5	Fatty Acid Binding Protein 5	–	0.795^***^	–	13	12	13	577.08	547.39	586.27
P63313	TMSB10	Thymosin Beta 10	–	0.794^***^	0.780^***^	2	2	2	78.77	50.13	88.21
Q9UBV8	PEF1	Penta-EF-Hand Domain Containing 1	0.791^***^	–	–	1	1	1	24.61	26.66	33.48
P15090	FABP4	Fatty Acid Binding Protein 4	–	0.789^***^	–	7	7	8	305.98	352.26	374.26
Q562R1	ACTBL2	Actin, Beta Like 2	0.760^**^	0.776^**^	–	1	3	2	328.7	432.69	370.57
P06703	S100A6	S100 Calcium Binding Protein A6S100A6	–	0.770^***^	–	3	3	5	92.85	92.68	110.27
P62328	TMSB4X	Thymosin Beta 4, X-Linked	–	0.760^***^	0.710^***^	2	2	1	79.31	67.49	75.61

*Pink indicates up-regulation; green indicates down-regulation; –, not significantly regulated*;

* *p < 0.05*;

** *p < 0.01*;

*** *p < 0.001*.

Several common proteins were identified that altered host cells among our three strains. Only one host protein, regulator complex protein LAMTOR2, was up-regulated by *M. bovis* and BCG infections but not by *H37Rv*. Four host proteins (three were up-regulated and only ACTBL2 was down-regulated) were differentially regulated by *H37Rv* and BCG infections and not by *M. bovis* infection. The three up-regulated host proteins were RPL19, RPL13, and MT-ND4. Nine host proteins (seven were up-regulated and two were down-regulated) were differentially regulated by *H37Rv* and *M. bovis* infections but not by BCG infection. These seven up-regulated host proteins included ICAM1, APOE, CCL20, KRT9, DERL1, SOD2, and NCF1. The two down-regulated proteins were TMSB4X and TMSB10.

Several proteins were differentially regulated by variant sources of mycobacteria. *M. bovis* infection induced the up-regulation of 19 proteins (including VPS26A, RBM17, REP15, CMTM6, YARS2, NDRG1, and RNF20), but *H37Rv* or BCG infection did not induce significant alterations in the levels of these proteins. Conversely, *H37Rv* infection induced the up-regulation of two interferon-induced proteins (IFIT1 and IFIT3) and the down-regulation of eight other proteins (including S100A6, FABP4, FABP5, GABARAPL2, and LGALS1), but *M. bovis* or BCG infection induced non-significant changes in the levels of these proteins. Meanwhile, five host proteins were differentially up-regulated (including ATP6V0C and SEC61A1), and only one was down-regulated (PEF1) by BCG infection but not by *H37Rv* or *M. bovis* infection.

### Functional ontology classification and bioinformatics analyses of differentially regulated proteins

**Figure 4:**
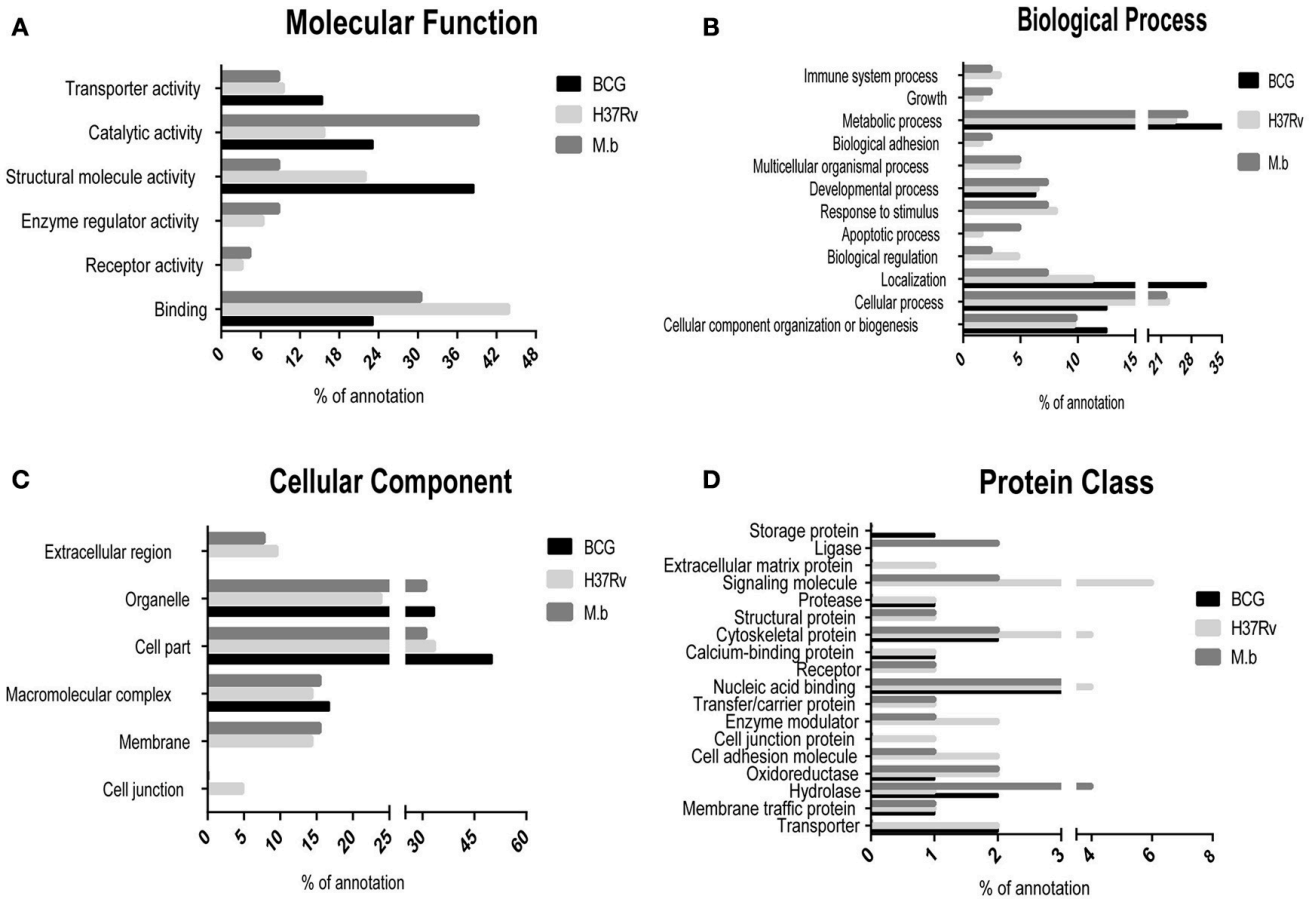
**Comparison of GO term annotation for significantly regulated proteins. (A)** Molecular Function, **(B)** biological process, **(C)** cellular component, and **(D)** protein class.

The differentially expressed proteins were cataloged into different molecular functions (Figure 4A), biological processes (Figure 4B), cellular components (Figure 4C), and protein class (Figure 4D) by searching the Gene Ontology and Uniprot databases. Differentially regulated *M. bovis*-infected cell proteins were found to mainly localize to metabolism, enzymatic activity, and cell part. Among 34 modulated proteins in the *H37Rv*-infected samples, these proteins were mainly implicated in metabolic processes, as well as *BCG*-induced host proteins. Together, the GO annotation comparison provided a comprehensive overview of differences regarding THP-1 infected with virulent *H37Rv* and *M. bovis* strains and the BCG vaccine strain, proteins which may be important in TB pathogenesis and transmission.

**Figure 5:**
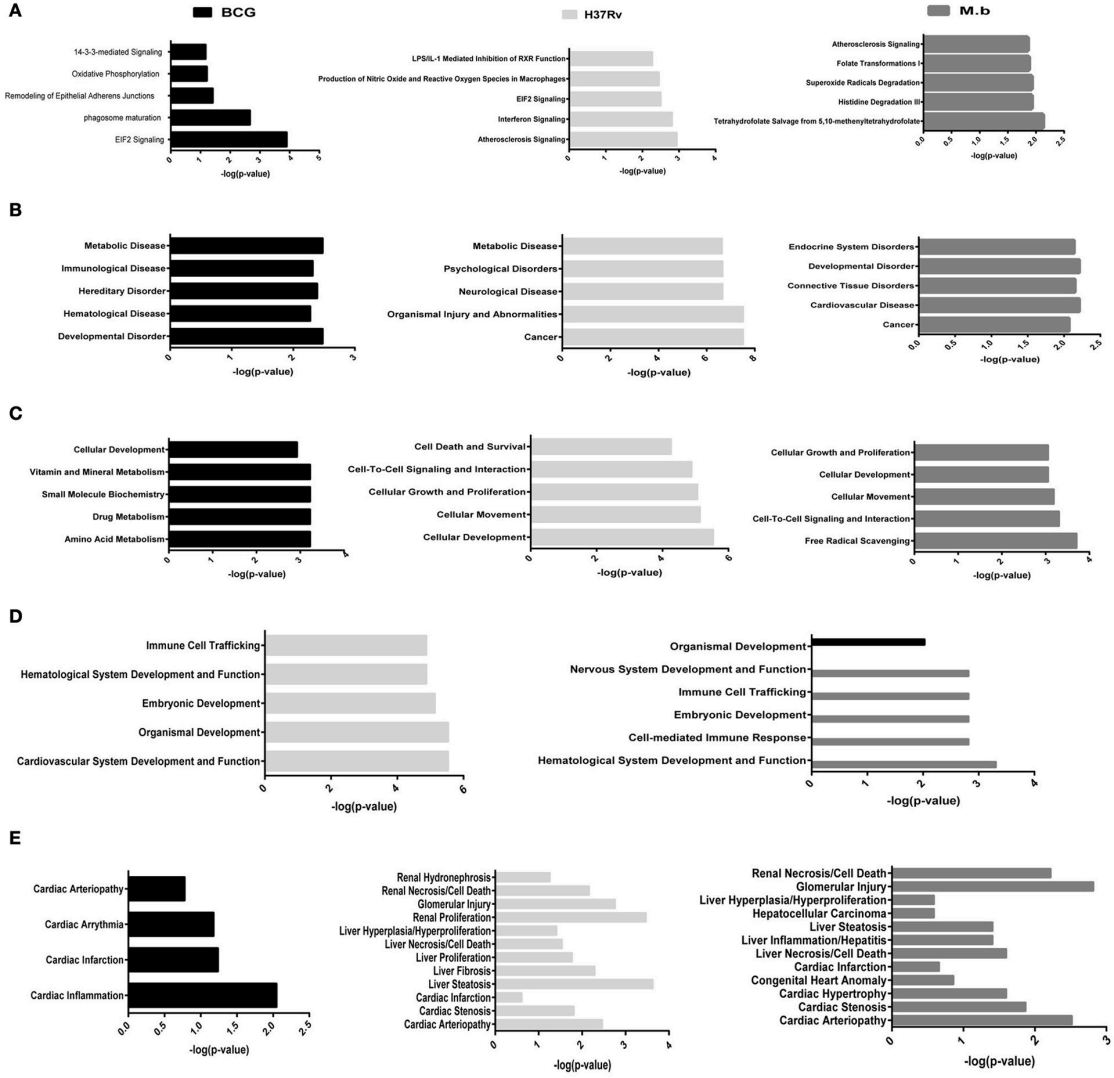
**Functional characterization of significantly altered proteins in THP-1 cells infected with MTBC strain ***H37Rv***, ***M. bovis***, or BCG. (A)** Top canonical pathways, **(B)** diseases and disorders, **(C)** molecular and cellular functions, **(D)** physiological system development and functions, and **(E)** toxicity functions.

To better analyze the function characterization and biological processes of relevant proteins, the Uniprot accession numbers and ratios (+Strain/Mock) of all differentially expressed proteins were uploaded into the IPA software. According to the function classification, all the differentially expressed proteins could be classified into five distinctive functional settings: (A) top canonical pathways; (B) diseases and disorders; (C) molecular and cellular functions; (D) physiological system development and functions; (E) toxicity Functions (shown in Figure 5 and Table S3, *p* < 0.05). These proteins were involved in various biological processes and functions, including cancer, immune cell trafficking, immunological disease, inflammatory disease, cell death and survival, protein trafficking, metabolic disease, cell-to-cell signaling and interaction, and infectious diseases. To note, immunological disease, as well as inflammatory response and disease, were more prominent upon infection of cells with virulent *H37Rv* and *M. bovis* than the BCG strain.

**Figure 6:**
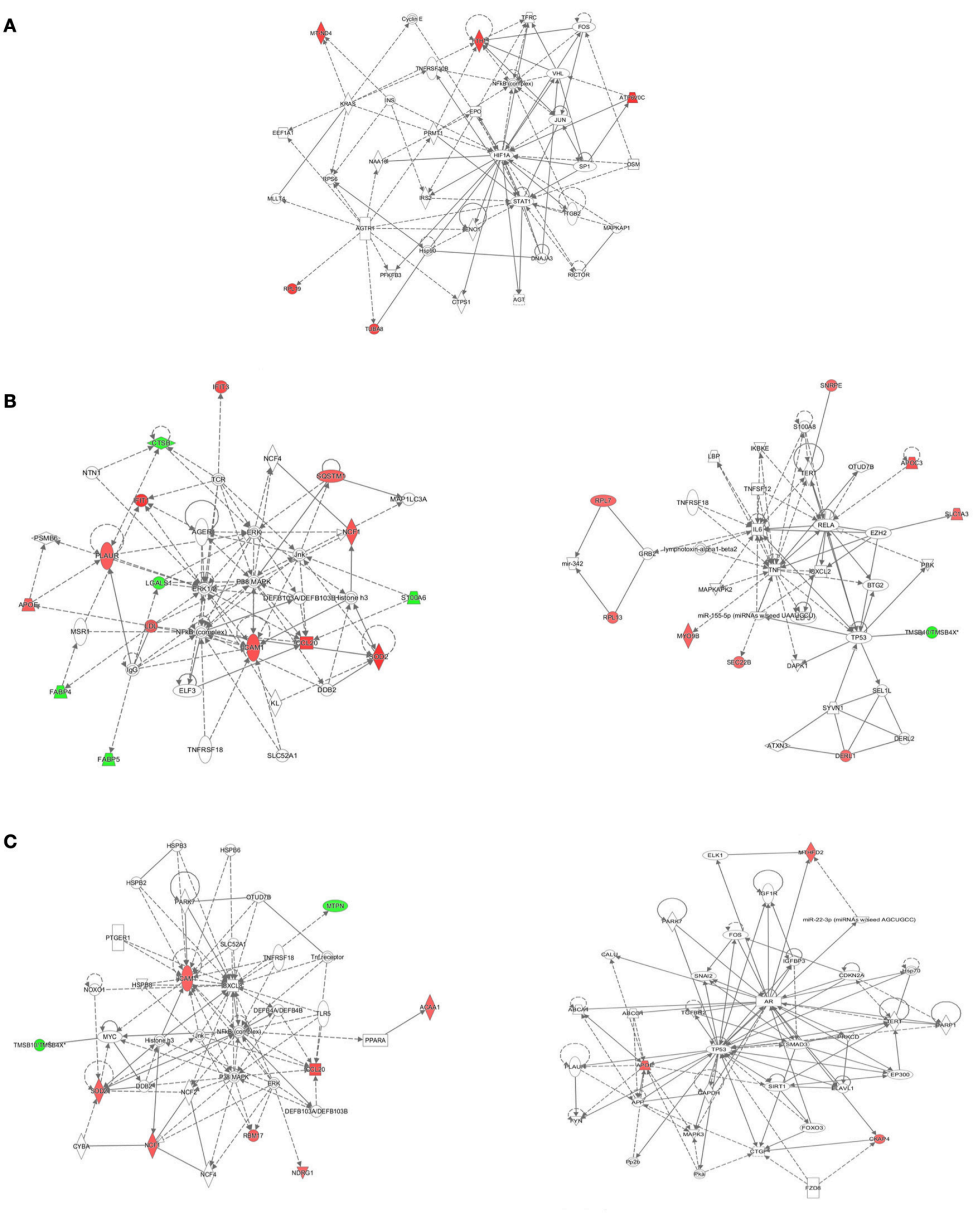
**The detailed view of the top-score networks**. Red indicates significantly up-regulated proteins, and green indicates significantly down-regulated proteins. White indicates the proteins that were not identified in our data. The color depth reflects the fold-change of proteins. The shapes are indicative of the molecular class. Lines with arrows connecting between the molecules indicate molecular relationships. Solid lines indicate direct interactions, and dashed lines indicate indirect interactions. **(A)** The top-score IPA network of BCG infection: cellular development, cellular growth and proliferation, cell death and survival, **(B)** the top two IPA networks of *H37Rv* infection: cellular movement, hematological system development and function, and immune cell trafficking (left); cancer, cell death, and survival, and cellular movement (right), **(C)** the top two IPA networks of *M. bovis* infection: free radical scavenging, hereditary disorder, and immunological disease (left); cell death and survival, organismal injury and abnormalities, and tissue morphology (right). Additional networks are depicted in Table S4.

The IPA tool was used to explore the potential, specific functional networks for proteins that changed in abundance. In total, 38 protein networks were mapped based on these differentially regulated proteins upon *M. bovis, H37Rv*, and BCG infection, respectively (Table S4). To investigate the underlying biologically functional differences that may be related to MTBC infection, five strongly represented networks of interest were depicted. For BCG infection, the top-score network was cellular development, cellular growth and proliferation, cell death and survival (5 molecules; score 11; Figure 6A). For *H37Rv* infection, the top two networks were: (1) cellular movement, hematological system development and function, and immune cell trafficking (14 molecules; score 29; Figure 6B, left); and (2) cancer, cell death, and survival, and cellular movement (9 molecules; score 16; Figure 6B, right). For *M. bovis* infection, the top two networks were: (1) free radical scavenging, hereditary disorder, and immunological disease (9 molecules; score 18; Figure 6C, left); (2) cell death and survival, organismal injury and abnormalities, and tissue morphology (3 molecules; score 5; Figure 6C, right). All networks showed significant differences in the specific members that were up-regulated, un-regulated, or down-regulated upon BCG, *H37Rv*, or *M. bovis* infection.

Similarly, IPA analysis identified numerous significantly-affected canonical pathways. The phagosome maturation pathway was differentially affected by BCG and *H37Rv* treatments (Figure S1A). Moreover, several proteins were similarly regulated by virulent *H37Rv* and *M. bovis*: differentially regulated proteins CCL20 and ICAM1, both involved in the TNF signaling pathway (Figure S1B); differentially regulated proteins NCF1 and ICAM1, both involved in the leukocyte transendothelial migration pathway (Figure S1C). Additional representative canonical pathways, such as EIF2 signaling, rheumatoid arthritis, PPAR signaling pathway, SNARE interactions in vesicular transport, peroxisome, and chemokine signaling pathway, were also differentially regulated upon infection (Table S5).

### String analysis

**Figure 7:**
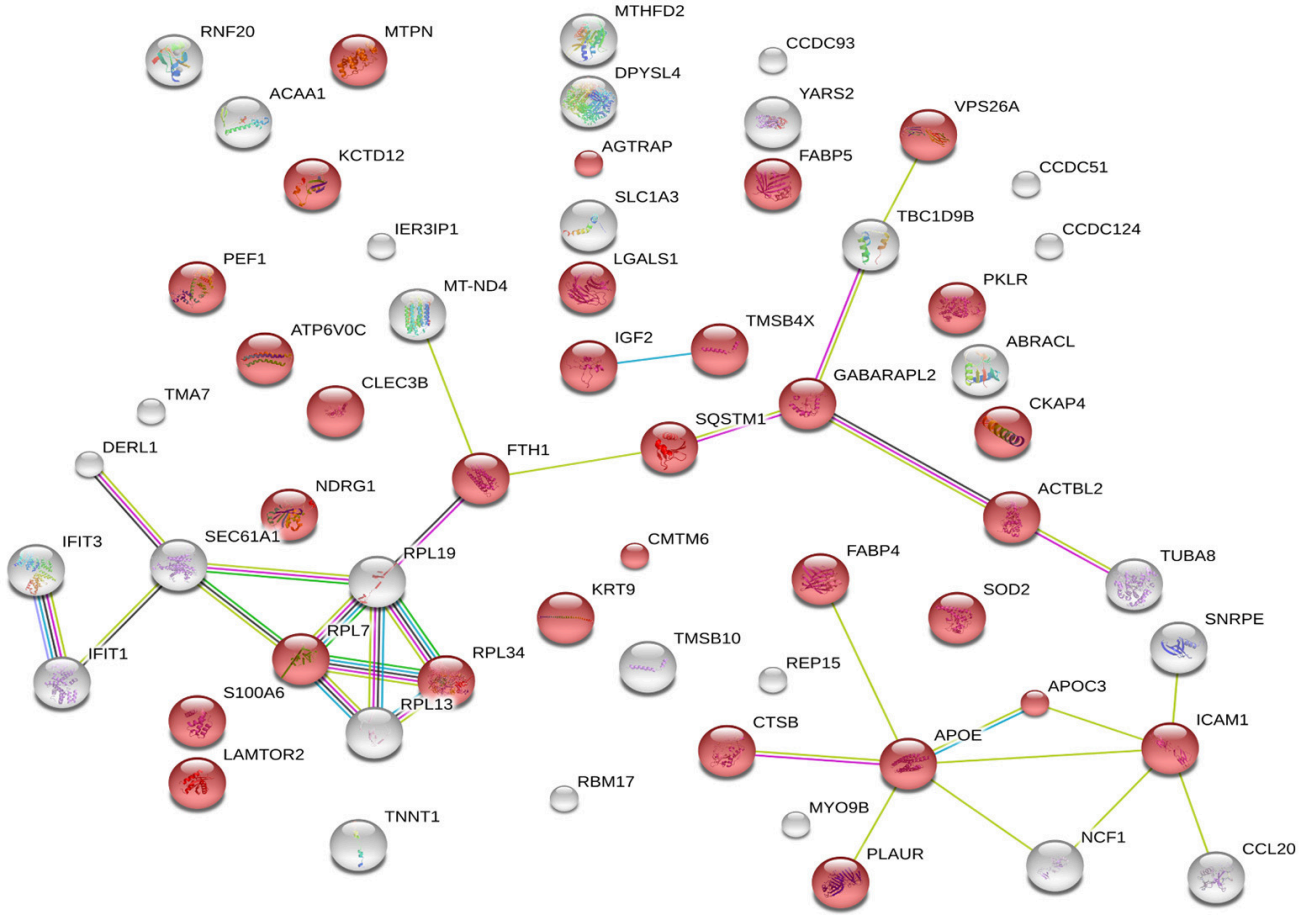
**STRING analysis revealing the interaction partners of the significantly altered proteins**. Red color indicates 31 proteins involved in vesicle category.

The 60 differently expressed proteins were analyzed using STRING-10 with a medium confidence score threshold of 0.4, and an interactome network was built for these set of proteins to find out protein-protein interaction and predict functional associations. We found that proteins involved in signaling pathway, migration pathway, vesicular transport, and intermediary metabolism interacted with each other as well as their partners; in addition, SEC22b cannot be founded by STRING-10. We also found that 31 proteins were involved in vesicle category and that most of them interacted with each other as well as their partners (Figure 7).

### Quantitative real-time (qRT)-PCR validation

**Figure 8:**
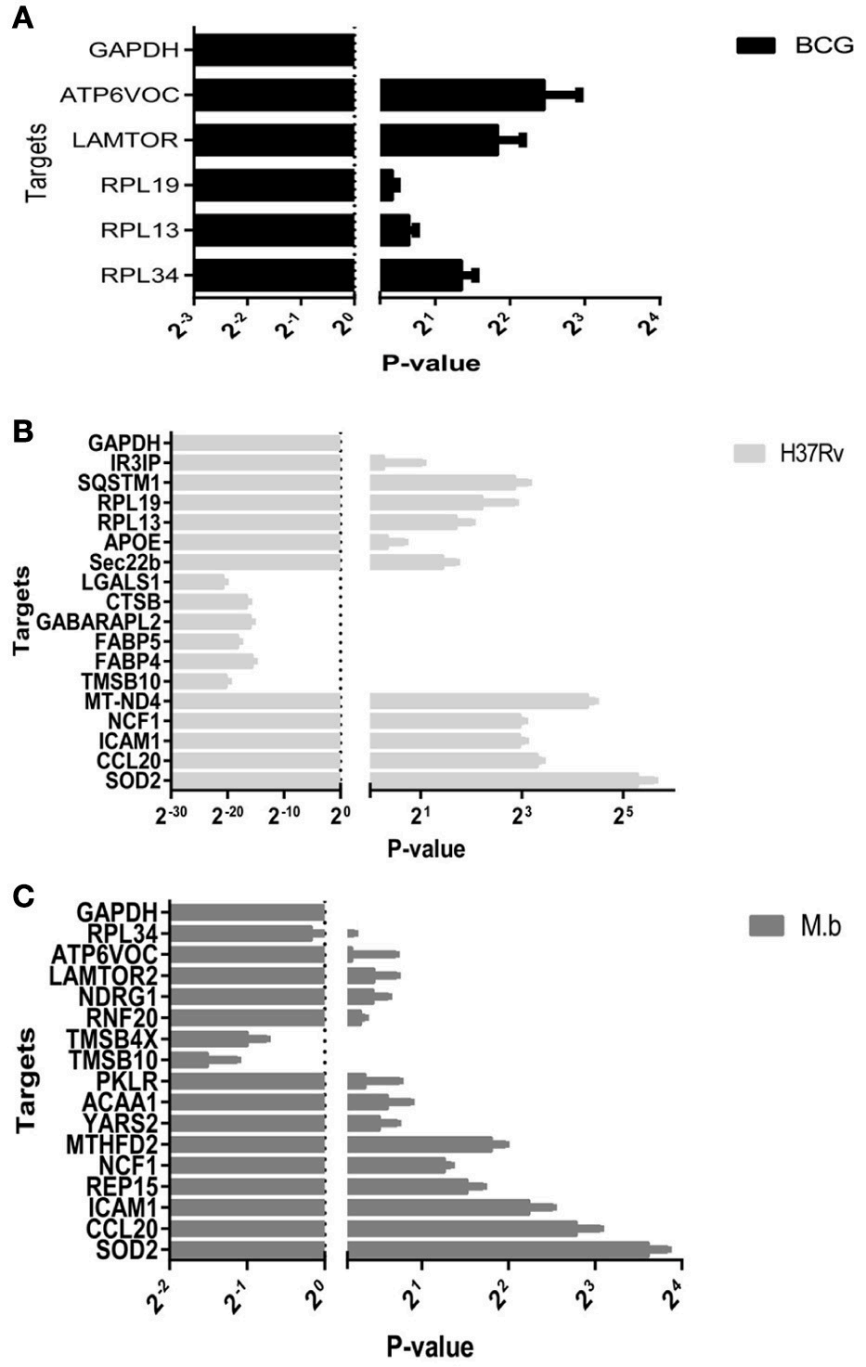
**Real-time RT-PCR analysis of significantly altered proteins in THP-1 cells infected by MTBC (10 MOI) or a mock. (A)** BCG in black bars, **(B)** *H37Rv* in gray bars, **(C)** *M. bovis* in dark gray bars.

To confirm the differential expression of the cellular proteome during MTBC-infection, 28 genes based on interest and different ratios were selected and assessed via qRT-PCR (Figure 8). The ratios from qRT-PCR analysis were in accordance with those obtained from the iTRAQ approach.

## Discussion

To date, the proteomics field has increasingly focused on intrinsically different proteins during host-pathogen interactions induced by MTBC infection. A variety of proteomic approaches have been applied to cells stimulated by infection (Rao et al., 2009; Li et al., 2011; Kaewseekhao et al., 2015; Saquib et al., 2015; Diaz et al., 2016; Long et al., 2016) or mycobacterial bioactive lipids (Shui et al., 2008). Comparisons of the results obtained in the current study with a previous proteomic assay of the BCG infected THP-1 cell (Lee et al., 2010) indicated that 392 common proteins and were identified and measured in both studies. There was good correlation between the two studies: 367 proteins were indicated as not significantly regulated in both assays and no proteins were indicated as significantly up-regulated in one assay but down-regulated in the other. Interestingly, lysosome-associated protein- LAMP-2 was tested as well as LAMTOR2 is discovered up- regulated in present BCG-infected cell study. There was no common protein comfirmed as significantly regulated in both studies, partly because the different time and method for purification of BCG phagosomes. Remarkably, differentially regulated proteins in these studies were associated with hemopoiesis, vesicle transport, apoptosis, phagosome maturation, immune cell trafficking, and the inflammatory response. Not all processes were affected by all MTBC strains or in different cell type, especially the virulent *M. bovis*-human host interaction. Thus, the current study was conducted to directly compare a live vaccine strain and two virulent strains, *H37Rv* and *M. bovis*, in a common cell type that had been previously used.

It is known that different MTB genetic groups can exhibit different features that affect protein extraction (Bespyatykh et al., 2016). The well-studied laboratory *H37Rv* strain cultured in cell culture media shows no net increase in viable bacilli numbers out to 7 days (Ackart et al., 2014). Because we used the same workflow both for three different strains, to be sure of its effectiveness we compared the number of identifed proteins within functional categories. It allows us to conclude that the differences in protein abundance we observed from MTBC-infection are independent from our workflow and reflect the true physiological characteristics of pathogens. Upon cellular infection, the two virulent strains induced host alterations were major differences in the proteins and pathways, comparing responses to mock and vaccine strain BCG. It was shown that SOD2, KTR9, CCL20, and ICAM1 were significantly up-regulated by *H37Rv* and *M. bovis* infections. Moreover, two of these proteins, CCL20 and ICAM1 which involved the TNF signaling pathway, were prominently upregulated at the mRNA level via qRT-PCR (Figure 8). The group of virulence *H37Rv* infection showed a higher dysregulation distribution of proteins across functional categories, in agreement with the results of Li et al. (2011).

**Figure 9:**
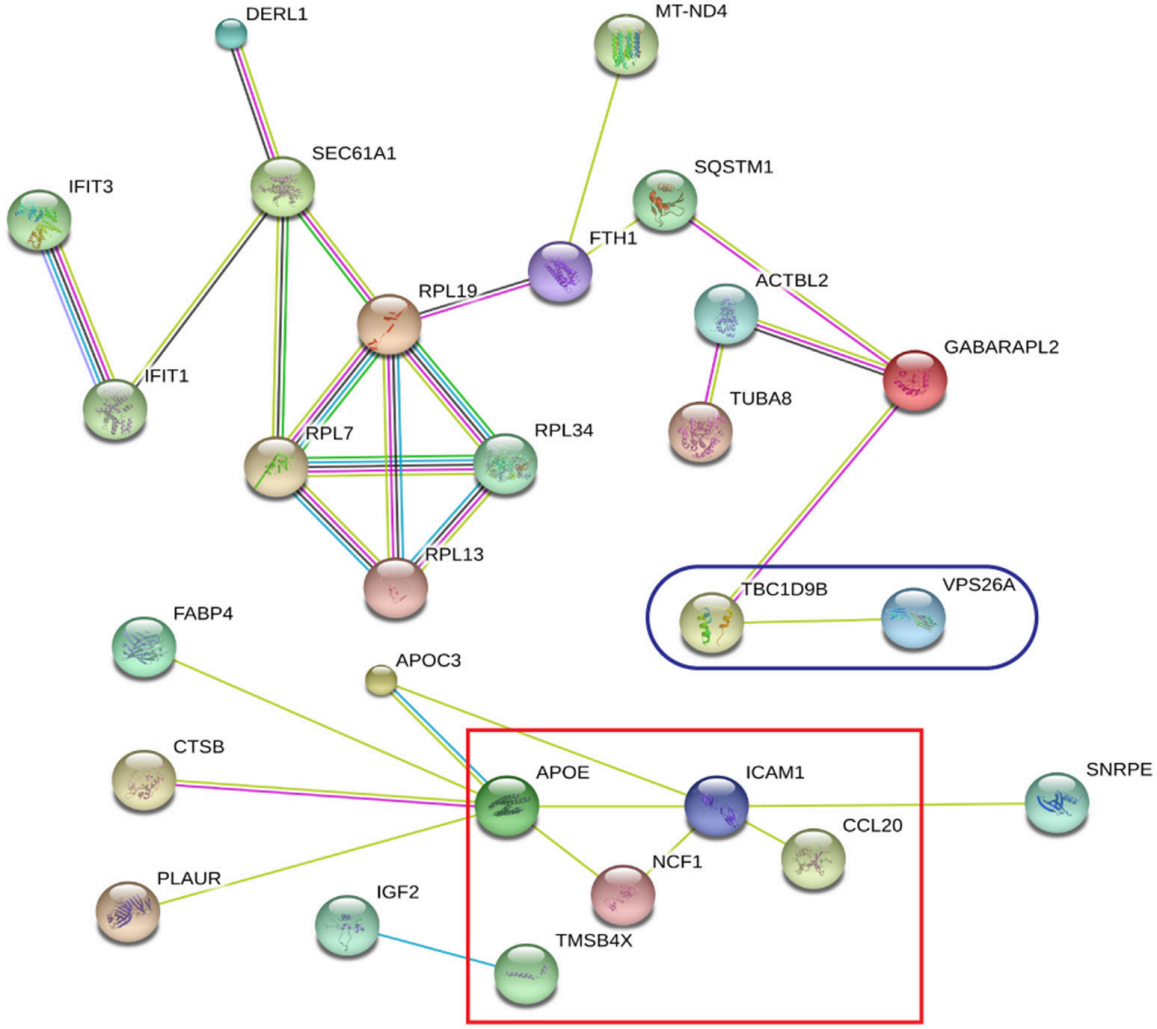
**STRING analysis revealing 27 interaction partners of the significantly altered proteins**. The blue oval indicates unique proteins involved in *M. bovis* induced proteins; the red square indicates the common proteins involved in virulent strains-*H37Rv* and *M. bovis* induced proteins.

Global protein analyses of the post translational modifications (PTMs) have deciphered that PTMs are associated with pathogenicity in bacteria, including phosphorylation (Calder et al., 2016), lysine succinylation (Yang et al., 2015), and glycosylation (Hare et al., 2017). It is striking that IFIT1 and IFIT3, two interferon-induced proteins involved in interferon signaling (Hare et al., 2015, 2017), showed significant up-regulation among the *H37Rv*-induced proteins. Moreover, several proteins involved in PTMs were differently regulated by *M. bovis*-infection, including RNF20, YARS2, and TMSB10 (Table 1). A study has demonstrated that LprG of *M. bovis* interacted with LAMP-3 to modulate the traffic machinery in cells (Vázquez et al., 2016). LAMTOR2 might be a potential target for endosome trafficking events of *M. bovis* by interacting with bacterial proteins. Since a small amount of enzyme can promote high efficiency response, the enzyme MTHFD2, SOD2, YARS2, and RNF20 were differentially regulated during *M. bovis* infection. Moreover, two of these proteins, SOD2 and MTHFD2 which involved in metabolism pathways, were prominently upregulated at the mRNA level via qRT-PCR (Figure 8). Particularly, the analysis of canonical pathways revealed that bifunctional methylenetetrahydrofolate dehydrogenase (MTHFD2) was involved in the pathways “tetrahydrofolate salvage from 5,10-methenyltetrahydrofolate, histidine degradation III, and folate transformations I” and can directly interact with each other (Figure 6C, right), suggesting that MTHFD2 enzyme is effective upon *M. bovis* infection. Among the 61 differently regulated proteins, the interactome network revealed that 27 proteins interacted with each other. The vacuolar protein VPS26A, involved in endocytosis, interacted with TBC1D9B uniquely induced by *M. bovis*. Meanwhile, five common proteins induced by virulence strains-*M. tb* and *M. bovis* involved in signaling pathway interacted with each other (Figure 9). Our findings have contributed to a large number of cellular proteins that are differentially regulated by *M. tb* and *M. bovis*.

The successful establishment and maintenance of bacterial infection not only depends on the pathogenic bacteria's ability but also the host cell's defense response. Different strains of the same species can also induce differences in pathology (Price et al., 2003; Jeon et al., 2008; Shah et al., 2013; Bai et al., 2015; Zhou et al., 2016). The present proteomic profiles of cellular proteins are beneficial to improving and enhancing understanding of the existing network of cross-talk mechanisms between the host and pathogen (Sambarey et al., 2013; Wu et al., 2014; Diaz et al., 2016; Li et al., 2016; Vázquez et al., 2016).

In conclusion, our study presents for the first application of proteomic analysis to compare whole cellular protein alterations induced by virulent *M. bovis* infections. The lysosomal adaptor LAMTOR2 might be a potential target for endosome trafficking events of *M. bovis*. The MTHFD2, VPS26A, and TBC1D9B proteins uniquely induced by virulent *M. bovis* infections might reveal novel biomarkers, which are also critical in cattle-to-human transmission and diagnosis of TB. Even though our fndings do not have an immediate proofs to elucidate the mechanism of how *M. bovis* interact with the host, they represent the proof-of-concept that virulent *M. bovis* will induce human cells with a characteristic proteome. To further characterize the intimate and persistent connection between these common mycobacterial pathogens, we will still need to molecularly explore the host-pathogen landscape, identify molecules with anti-TB function, and determine the means to clinically treat based on insight gleaned from pathogen-host interactions at both a global and a targeted level.

## Author contributions

CT and HC designed experiments, PL wrote the manuscript. PL, RW, WD, LH, BZ and YZ performed the experiments. All authors analyzed and interpreted data. YX, AZ, AG, and XW participated in the interpretation and discussion of the results. All authors read and approved the final manuscript.

## Funding

This work was supported by grants from the National Natural Science Foundation of China (NSFC) (No. 31421064), the National Key Research and Development Program of China and the Hubei Province Natural Science Foundation for Innovative Research Groups (2016CFA015).

### Conflict of interest statement

The authors declare that the research was conducted in the absence of any commercial or financial relationships that could be construed as a potential conflict of interest.

## Supplementary material

Figure S1 **Significantly affected canonical pathways determined by IPA® analysis**. Red indicates up-regulated proteins, light green indicates down-regulated proteins, gray represents not significantly affected proteins, and white represents proteins known to be part of the pathways but not identified by iTRAQ. **(A)** The phagosome maturation pathway is differentially affected by BCG and *H37Rv* treatments, **(B)** the TNF signaling pathway is similarly regulated by virulent *H37Rv* and *M. bovis*, **(C)** the leukocyte transendothelial migration pathway is similarly regulated by virulent *H37Rv* and *M. bovis*. Examples of additional affected canonical pathways are depicted in Table S5.

Table S1 **List of real-time RT-PCR primers used in this study**.

Table S2 **List of the 2,032 unique proteins detected our study**. Exp pr, three-experiment *p*-value; Group pr, group *p*-value.

Table S3 **Functional classification of 61 differentially expressed proteins**.

Table S4 **Network analysis of 38 differentially expressed proteins**.

Table S5 **Canonical pathway analysis of differentially expressed proteins**.
