# Supplementary figures and images for: Comparative Proteomics Analysis of Human Macrophages Infected with Virulent Mycobacterium bovis
Source: Front Cell Infect Microbiol. 2017 Mar 9;7:65. doi: 10.3389/fcimb.2017.00065 (PMC5343028; doi:10.3389/fcimb.2017.00065)

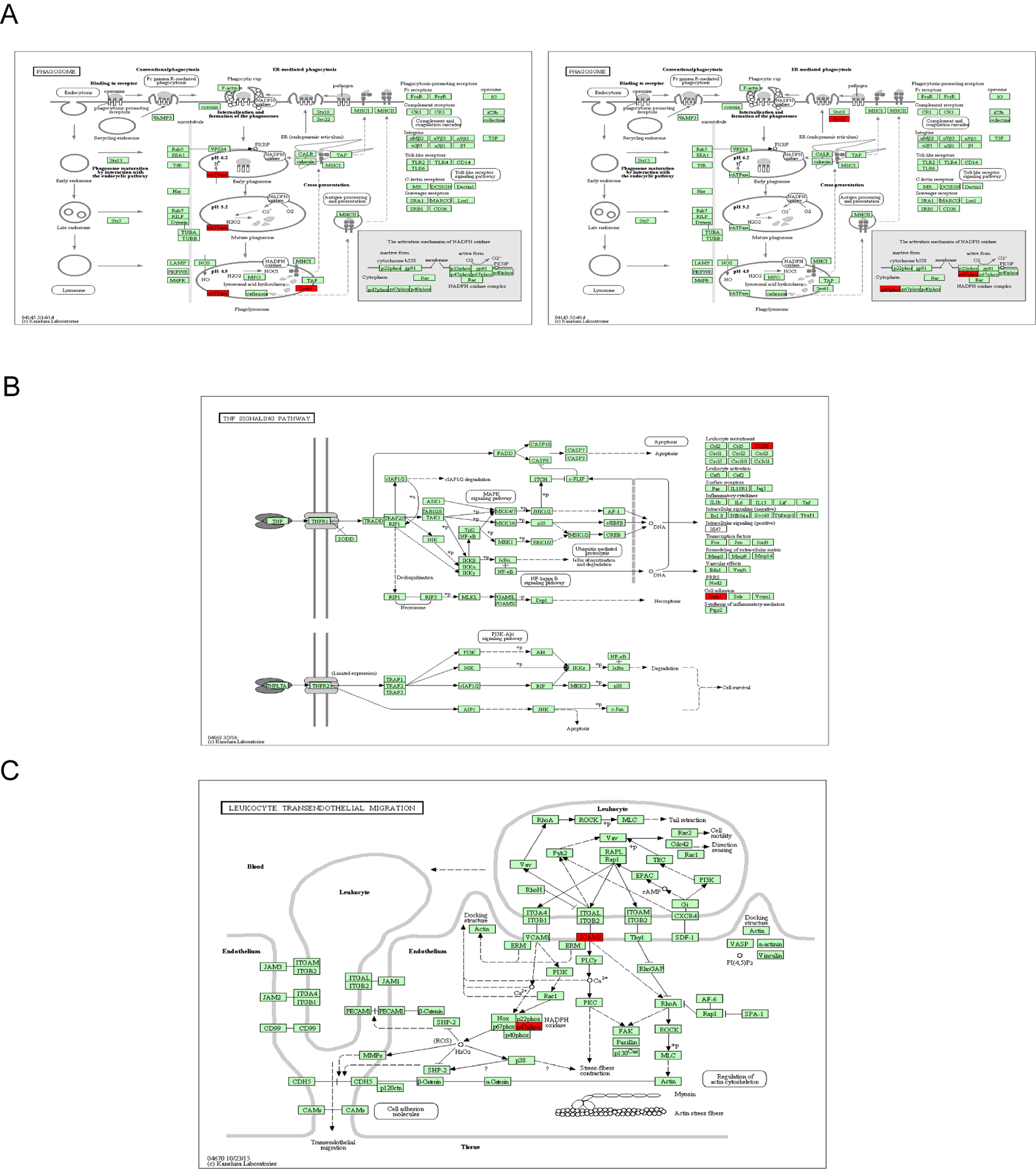

Supplement: Figure S1 — Significantly affected canonical pathways determined by IPA® analysis. Red indicates up-regulated proteins, light green indicates down-regulated proteins, gray represents not significantly affected proteins, and white represents proteins known to be part of the pathways but not identified by iTRAQ. (A) The phagosome maturation pathway is differentially affected by BCG and H37Rv treatments, (B) the TNF signaling pathway is similarly regulated by virulent H37Rv and M. bovis, (C) the leukocyte transendothelial migration pathway is similarly regulated by virulent H37Rv and M. bovis. Examples of additional affected canonical pathways are depicted in Table S5. [file Image1.TIF]
