# Supplementary material for: Comparative Proteomics Analysis of Human Macrophages Infected with Virulent Mycobacterium bovis
Source: Front Cell Infect Microbiol. 2017 Mar 9;7:65. doi: 10.3389/fcimb.2017.00065 (PMC5343028; doi:10.3389/fcimb.2017.00065)
Supplement: Table S1 — List of real-time RT-PCR primers used in this study. [file Table1.DOC]

**Supplementary Table S1. List of real-time RT-PCR primers.**

| ID | Gene Symbol(s) | Sequence 5'-3' |
| --- | --- | --- |
| P04179 | SOD2 | CCGAGGAGAAGTACCAGGAG |
|  |  | GATTGATATGACCACCACCATTGA |
| P78556 | CCL20 | GAAGGCTGTGACATCAATGCTAT |
|  |  | GGTTCTTTCTGTTCTTGGGCTAT |
| P05362 | ICAM1 | ACCTATGGCAACGACTCCTT |
|  |  | AGTGTCTCCTGGCTCTGG |
| P14598 | NCF1 | CGCCATTGCCAACTACGA |
|  |  | GAACCACCAACCGCTCTC |
| P03905 | MT-ND4 | TACGCCTCACACTCATTCTCA |
|  |  | TATGTGGCTGATTGAAGAGTATGC |
| P63313 | TMSB10 | ACGGAGACGCAGGAGAAG |
|  |  | GTGGCTCGTGTCCATCTTG |
| P15090 | FABP4 | AGTCAAGAGCACCATAACCTTAGA |
|  |  | GACGCATTCCACCACCAG |
| Q01469 | FABP5 | ATTGGTTCAGCATCAGGAGTG |
|  |  | ATAGATCCGAGTACAGGTGACATT |
| P60520 | GABARAPL2 | AAGTGAGGTAGGTGCGGTATT |
|  |  | CAAGTGTTCTGACTGCTAAGGATT |
| P07858 | CTSB | CAAGGAATGCCTGTGCCAATA |
|  |  | GCAGCGAGAAGTTAAGATGAAGT |
| P09382 | LGALS1 | GTGGCTCCTGACGCTAAGA |
|  |  | AGGCTGGAAGGGAAAGACA |
| O75396 | Sec22b | GAAGCACTCTCAGCATTGGATT |
|  |  | AGCCACCAGAATCGGACATA |
| P02649 | APOE | GCCCTATAATTGGACAAGTCTGG |
|  |  | CCTTCATCTTCCTGCCTGTG |
| P26373 | RPL13 | TGGAATCAGTCGGCAGTCA |
|  |  | TGGCAGGAGGAAGTCACA |
| P62328 | TMSB4X | AGAAGACAGAGACGCAAGAGA |
|  |  | CGCACGCCTCATTACGATT |
| P84098 | RPL19 | CGGAGCGAACAAGACCAAG |
|  |  | CCACAGCGGAGGACACTA |
| Q13501 | SQSTM1 | TCGGATAACTGTTCAGGAGGAG |
|  |  | CTTCGGATTCTGGCATCTGTAG |
| Q9Y5U9 | IR3IP | CACAAGAGCAGTCCTAAACAATCA |
|  |  | ACCTATCCAGCATCAATGTCCTA |
| Q6BDI9 | REP15 | CCACCACCAAGATGACCAAG |
|  |  | CACAAGGCTTAGATTCCACAGG |
| P30613 | PKLR | CGCCAGGTCCACTTATGC |
|  |  | GCTTTCCACTTTCAATGCCAAAT |
| Q9Y2Z4 | YARS2 | GCTGCTTTCCTATCTTACCCTTC |
|  |  | GCCTCACAATTCCTTACACTACC |
| P09110 | ACAA1 | AGGTCCAAGGCAGAAGAGTT |
|  |  | GGCATAGGCAGGTCCAATG |
| P13995 | MTHFD2 | GCGGCAGGAGGTAGAAGA |
|  |  | TTGTTGAGGACATAGGAGTGACT |
| Q92597 | NDRG1 | CGGAAGGCTGGATGGACT |
|  |  | CAATGTGCTGGCGGTAGG |
| Q9Y2Q5 | LAMTOR2 | AACCTTCTGCTGTGTATGTATGC |
|  |  | GCTTCCACCAATGCCGTTA |
| Q5VTR2 | RNF20 | TCAGTAGCCTCCAGAATCACAA |
|  |  | CATCCTTCGGGTCCTCAGT |
| P27449 | ATP6V0C | GAACGAACAGCCTGACACAT |
|  |  | CCGCATACACAGAGCACTC |
| P49207 | RPL34 | TTCGTGCTGTAAGACCTAAAGTTC |
|  |  | CTCTGTGCTTGTGCCTTCAA |
| P04406 | GAPDH | TCATGACCACAGTCCATGCC |
|  |  | ggatgaccttgcccacagcc |
